# Risk factors for empty follicle syndrome in diminished ovarian reserve patients undergoing intracytoplasmic sperm injection cycles: A retrospective observational analysis

**DOI:** 10.1097/MD.0000000000038902

**Published:** 2024-07-12

**Authors:** Belgin Devranoğlu, Müşerref Banu Yilmaz, Gamze Peker, Özlen Emekçi Özay, Ali Cenk Özay, Ali İrfan Güzel

**Affiliations:** a Zeynep Kamil Women and Children’s Diseases Training and Research Hospital, Health Sciences University, İstanbul, Turkey; b Department of Obstetrics and Gynecology, Ümraniye Training and Research Hospital, İstanbul, Turkey; c Department of Obstetrics and Gynecology, Cyprus International University, Nicosia, Cyprus; d Department of Obstetrics and Gynecology, Sanko University, Gaziantep, Turkey.

**Keywords:** diminished ovarian reserve, empty follicle syndrome, risk factors

## Abstract

The aim of this study is to evaluate the risk factors for empty follicle syndrome (EFS) in patients with diminished ovarian reserve (DOR) undergoing an intracytoplasmic sperm injection cycle. In this retrospective study, patients with DOR were divided into 2 groups according to the presence of empty follicles on the day of oocyte retrieval. Patient age, body mass index (BMI), anti-Müllerian hormone (AMH), baseline follicle stimulating hormone (FSH) and estradiol (E2) levels, basal antral follicle count (AFC), total gonadotropin dose, and day of stimulation were recorded as risk factors. The association between EFS and these variables was assessed using the logistic regression method and ROC curve analysis. Increased BMI, low AMH, higher baseline FSH, low baseline AFC, higher gonadotropin dose, and longer day of ovulation induction were independent risk factors for EFS in patients with DOR. ROC curve analysis showed that BMI, AMH, baseline FSH, baseline AFC, higher gonadotropin dose, and longer ovulation induction days were predictive parameters in this group. According to the current study, higher BMI, lower AMH, higher baseline FSH, lower baseline AFC, higher gonadotropin dose and longer ovulation induction days were independent risk factors for EFS in patients with reduced ovarian reserve.

## 1. Introduction

Empty follicle syndrome (EFS) is a condition in which no eggs are retrieved from mature follicles despite obvious ovarian stimulation during in vitro fertilization treatment. The exact causes of EFS are not yet fully understood, but there are several potential risk factors that may contribute to its occurrence.^[1]^ It was first reported by Coulam et al^[2]^ in 1986. EFS is also a rare occurrence in assisted reproductive technology cycles. The incidence of EFS is estimated at 0.6% to 7.0%; the economic consequences and emotional frustration are enormous.^[3–6]^

Previous studies have shown some risk factors for EFS, such as advanced age, longer duration of infertility, higher baseline follicle stimulating hormone (FSH) levels and lower estradiol (E2) levels before human chorionic gonadotropin (hCG) injection.^[5]^ Kim and Jee^[7]^ also reported that decreased ovarian reserve due to ovarian aging could be a possible etiologic factor for EFS.

In the current study, we investigated the risk factors for empty follicle syndrome in patients with diminished ovarian reserve (DOR) undergoing an intracytoplasmic sperm injection (ICSI) cycle.

## 2. Methods

### 2.1. Study design

This was a single-center retrospective cohort study designed to evaluate differences in cycle abortions and clinical pregnancy rates among poor responders classified by age, ovarian reserve testing, and previous poor response.

### 2.2. Setting

The demographic and clinical information was taken from the data recorded during ICSI cycles performed between January 1, 2014 and March 1, 2018 at the Centre for Reproductive Medicine, Health Sciences University, Zeynep Kamil Women and Children’s Health Training and Research Hospital. Approximately 2000 cycles/yr are performed in our center according to institutional protocols that closely follow the recently published evidence.

### 2.3. Ethical statement

The study was approved by the Ethics Committee of Health Sciences University, Zeynep Kamil Women and Children’s Health Training and Research Hospital and was conducted in accordance with the Declaration of Helsinki.

### 2.4. Participants

Diminished ovarian reserve was defined by the following criteria: a basal FSH level > 10 IU/L, an antral follicle count <6 or a previous poor ovarian response. The presence of at least one of these criteria was required to make the diagnosis of poor ovarian reserve. Seven hundred sixty-two women with poor ovarian reserve, who were identified using the criteria described above and underwent ovarian hyperstimulation were included in the study. Individualized gonadotropin doses and protocols were selected according to our institutional conventional ovarian hyperstimulation protocols for patients with poor ovarian reserve. Patient data were retrospectively retrieved from the hospital database. Assisted reproductive technology outcomes were then compared for each group defined on the basis of individual classification systems. As there is no evidence to support a particular approach in this patient population, patients were assigned to a treatment protocol at the discretion of the treating physician.

*Inclusion criteria:* Patients were included in the study group if they had no oocyte on oocyte pick-up (OPU) day despite abundant follicle development and elevated E2 levels (n = 118); patients with even 1 or more oocytes on OPU day formed the control group (n = 644).

*Exclusion criteria:* Patients who had normal ovarian reserve, polycystic ovary syndrome, endocrine disorders and using hormonal therapy.

The number of antral follicles was measured on days 2 to 4 of the previous cycle. An antagonist protocol was used in all cases. Treatment was initiated on day 2 of the menstrual cycle with stimulation by recombinant FSH (Gonal-f, Merck-Serono, Geneva, Switzerland) at a daily dose of 300 to 450 IU. Follicular growth was monitored by transvaginal sonography. The dose of recombinant FSH was adjusted from day 5 of stimulation according to the ovarian response.

### 2.5. Ovulation induction method and oocyte retrieval

A daily injection of 0.25 mg of the antagonist (Cetrorelix, Merck-Serono, Geneva, Switzerland) was added to treatment when the leading follicle is ≥12mm. After a follicle size of 18 mm was reached, recombinant human chorionic gonadotropin (hCG) 250 μg was administered, followed by follicular puncture 36 hours after recombinant hCG administration under transvaginal ultrasound guidance. The luteal phase was supplemented twice daily with 8% intravaginal progesterone gel. The embryos were transferred on day 3 or 5 of each cycle. Two weeks after transfer, serum beta-hCG was measured. The quality of the embryos was assessed according to the criteria of the Istanbul Consensus Workshop.^[8]^ On day 3, embryos that reached the 6-cell stage with a fragmentation of 20% were classified as good quality embryos, while embryos that reached the 7-cell stage with a maximum fragmentation of 10% were considered best quality embryos.

### 2.6. Data assessment

The following risk factors were analyzed; patient age, body mass index (BMI; BMI = weight [kg]/height [m]^2^ and BMI > 30 was defined as obesity), baseline AMH, FSH, E2, baseline AFC, total gonadotropin dose and duration of ovarian stimulation.

### 2.7. Statistics

Statistical analyses were undertaken with Statistical Package for Social Sciences for Windows 17.0 (SPSS Inc., Chicago, IL). The Kolmogorov–Smirnov test was used to establish whether or not numeric data exhibited a normal distribution, and the percentage was expressed as mean ± standard deviation. Data demonstrating a normal distribution were analyzed with the student *t* test. The sample size was determined according to the results of the central limit theorem,^[9]^ which indicated that at least 30 individuals were required in each subgroup. Logistic regression analysis was used to find out the independent risk factors and ROC curve was used to define the discriminative power of these parameters. For the results thus obtained, 95% confidence interval and *P* < .05 were regarded as preconditions for statistical significance.

## 3. Results

A total of 762 participants’ results were analyzed. Table 1 shows the demographic and clinical characteristics of the groups. BMI was statistically significantly higher in the EFS groups (*P* < .05). The mean age of the patients did not differ statistically significantly between the groups (*P* > .05). The baseline levels of AMH and AFC were lower in the study group. Baseline FSH and E2 levels were higher in the study group (*P* < .05). The total gonadotropin dose and the days of ovarian stimulation were also higher in the study group (*P* < .05).

**Table 1:** Comparison of demographic and clinical features of patients in EFS and non-EFS group.

	Non-EFS group (n = 644)	EFS group (n = 118)	*P*
Age (yr)	35.7 ± 4.82	35.8 ± 4.72	.937
BMI (kg/m^2^)	27.3 ± 5.00	28.4 ± 4.78	**.021**
AMH (ng/mL)	0.47 ± 0.43	0.38 ± 0.33	**.031**
Baseline FSH (mIU/mL)	11.9 ± 7.77	14.8 ± 8.92	**.001**
Baseline estradiol (pg/mL)	57.6 ± 6.55	68.9 ± 7.01	**.042**
AFC	4.8 ± 1.25	3.4 ± 1.86	**.031**
Total gonadotropin doze (IU)	3698.8 ± 1131.93	4030.4 ± 1544.40	.0
Day of stimulation	9.1 ± 2.18	10.7 ± 2.90	.071

Bold values are statistically significant values.

AFC = antral follicle count, AMH = anti-Müllerian hormone, BMI = body mass index, EFS = empty follicle syndrome, FSH = follicle stimulating hormone.

**Figure 1.**
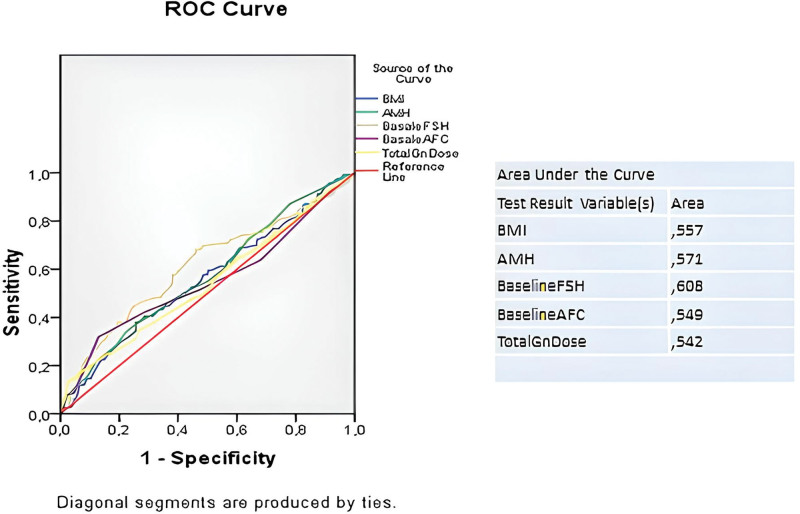
ROC curve analysis of discriminating risk factors.

The logistic regression method showed that higher BMI, lower AMH, higher baseline FSH, lower AFC and higher total gonadotropin dose were risk factors for EFS in patients with DOR (Table 2). ROC curve analysis revealed that BMI, AMH, baseline FSH, AFC, and total gonadotropin dose were discriminating factors for EFS in these patients (Fig. 1).

**Table 2:** Risk factors for empty follicle syndrome among obese patients.

	β	SE	Wald	Odds ratio	*P*
BMI (kg/m^2^)	0.050	0.210	5.497	1.051	**.019**
AMH (ng/mL)	0.526	0.253	4.329	1.692	**.037**
Baseline FSH (mIU/mL)	0.023	0.011	4.138	1.024	**.042**
AFC	-0.305	0.077	15.792	0.737	**.005**
Total gonadotropin dose	0.016	0.022	0.012	1.102	**.044**

Bold values are statistically significant values.

AFC = antral follicle count, AMH = anti-Müllerian hormone, BMI = body mass index, FSH = follicle stimulating hormone.

## 4. Discussion

In the current study, we identified the risk factors for EFS in patients with reduced ovarian reserve who underwent a cycle of ICSI. A total of 762 patients were included in this study. Of all patients, 118 patients had no follicle after OPU and were included in the study group. These patients had a statistically significantly higher BMI, a higher baseline FSH level and a higher total gonadotropin dose as well as a lower AMH level and also a lower AFC. Also, in this study we found that 15.5% of DOR patients had EFS.

Empty follicle syndrome is the absence of oocytes at the time of retrieval despite aspiration and careful flushing of the follicles. After using pituitary suppression protocols, the incidence decreases, but it is still very distressing for both the infertile couple and the clinical staff, especially in terms of counseling about future outcomes.^[2,10,11]^

In a previous study, Mitsui et al^[12]^ reported that patient age, BMI, and serum AMH level were not risk factors for EFS. They also found AFC and the number of dominant follicles before OPU to be risk factors.^[12]^ Similar to this study, we also found that patient age was not a risk factor for EFS; in contrast, we found BMI and AMH to be risk factors.

The effects of obesity on oocyte quality were described in a previous review by Erel and Senturk. ^[13]^ According to this review, obesity affects oocyte quality in terms of a lower number of oocyte retrievals, poorer oocyte quality or maturity, lower fertilization rate and lower embryo quality.^[13]^ Similar to this review, we also found in our study that obesity leads to EFS.

The association between EFS and AMH has been investigated in only a few studies, both of which reported that AFC was a better predictor of EFS than AMH.^[14,15]^ Mitsui et al^[12]^ also found a significant difference in their EFS group, but in a multivariate analysis they found that AMH was not a risk factor for EFS. In contrast to this study, we found lower AMH as a risk factor for EFS.

Madani and Jahangiri^[16]^ conducted a retrospective study that included 3356 cycles of in vitro fertilization. In this study, EFS was correlated positively with serum AMH level and negatively with baseline serum FSH level.^[16]^ Also, they suggested that EFS could be a sign of low ovarian reserve.^[16]^ Similar to this study, we found that patients with EFS had lower AMH and higher FSH levels. We also believe that the risk of EFS increases with the degree of DOR.

The limitation of this study is that it is retrospective. In addition, its most important strength is that it is one of the studies with the highest participation in the literature examining EFS, especially in patients diagnosed with DOR.

In conclusion, higher BMI, lower AMH and higher baseline FSH levels, lower baseline AFC, higher gonadotropin dose, and longer days for ovarian stimulation are independent risk factors for EFS in patients with DOR. Patients with DOR should attend an obesity control appointment and have a well-designed baseline hormone assessment and AFC measurement performed by an experienced clinician.

## Author contributions

**Conceptualization:** Muserref Banu Yilmaz.

**Data curation:** Belgin Devranoğlu, Muserref Banu Yilmaz, Ozlen Emekci Ozay, Ali Cenk Ozay.

**Formal analysis:** Belgin Devranoğlu, Ozlen Emekci Ozay, Ali Cenk Ozay, Ali Irfan Guzel.

**Methodology:** Gamze Peker, Ali Irfan Guzel.

**Writing – original draft:** Gamze Peker.

**Writing – review & editing:** Belgin Devranoğlu, Ali Irfan Guzel.
